# Postharvest Treatments with Sulfur-Containing Food Additives to Control Major Fungal Pathogens of Stone Fruits

**DOI:** 10.3390/foods10092115

**Published:** 2021-09-07

**Authors:** Victoria Martínez-Blay, Verònica Taberner, María B. Pérez-Gago, Lluís Palou

**Affiliations:** Centre de Tecnologia Postcollita (CTP), Institut Valencià d’Investigacions Agràries (IVIA), 46113 Montcada, Valencia, Spain; martinez_vicbla@externos.gva.es (V.M.-B.); taberner_ver@gva.es (V.T.); perez_mbe@gva.es (M.B.P.-G.)

**Keywords:** antifungal activity, GRAS salts, nectarines, *Geotrichum candidum*, *Monilinia fructicola*, *Rhizopus stolonifer*

## Abstract

The sulfur-containing salts, classified as food additives, sodium metabisulfite (SMBS), potassium metabisulfite (PMBS), aluminum sulfate (AlS), and aluminum potassium sulfate (AlPS), were evaluated for their activity against *Monilinia fructicola*, *Rhizopus stolonifer*, and *Geotrichum candidum*, the most economically important fungal pathogens causing postharvest disease of stone fruit. In in vitro tests with potato dextrose agar (PDA) Petri dishes amended with different concentrations of the salts (0, 10, 20, 30, 50, and 100 mM), SMBS and PMBS at all concentrations, AlS above 20 mM, and AlPS above 30 mM, completely inhibited the mycelial growth of the three fungi after incubation at 25 °C for up to 10 days. In in vivo primary screenings with artificially inoculated nectarines, aqueous solutions of the four salts reduced the incidence and severity of brown rot (BR) at concentrations of 10 and 50 mM, whereas only AlS and AlPS reduced Rhizopus rot (RR), and none of the salts was effective against sour rot (SR). Solutions at 100 mM were phytotoxic and injured the fruit peel. In small-scale trials, 1 min dip treatments at 20 °C in SMBS or PMBS at 10 mM significantly reduced the incidence and severity of BR after incubation at 20 °C for up to 8 days. Conversely, dips in AlS and AlPS reduced neither BR nor RR. Results highlight the potential of SMBS and PMBS as new nonpolluting tools for the integrated control of BR, but not RR and SR, on stone fruit.

## 1. Introduction

About 45 million tons of stone fruits are produced annually in temperate regions of the world, and the main economically important cultivated species include peaches, nectarines, plums, cherries, and apricots [1]. Stone fruits are highly perishable and characterized by a relatively short postharvest life. Fruit losses after harvest are due to physiological disorders of abiotic origin and especially to biotic diseases mainly caused by fungal pathogens. These diseases represent an important problem for both the fresh and the processing market [2]. Primary postharvest decay pathogens of stone fruit worldwide are *Monilinia* spp., especially *M. fructicola* (G. Wint.) Honey, *M. laxa* (Aderh. & Ruhland) Honey, and *M. fructigena* (Aderh. & Ruhland) Honey, all causing brown rot (BR); *Botrytis cinerea* Pers.:Fr., causing gray mold; *Rhizopus stolonifer* (Ehrenb.:Fr.) Vuill., causing soft rot or Rhizopus rot (RR); *Geotrichum candidum* Link, causing sour rot (SR); and *Penicillium expansum* Link, causing blue mold [3]. These fungi cause postharvest decay either by entering the fruit through peel wounds or injuries or after a period of latency that follows field infections of flowers or young fruit [4].

Current effective stone fruit postharvest disease control, and particularly control of postharvest BR caused by *Monilinia* spp., consists of an integrated combination of pre- and postharvest fungicide applications, sanitation practices, and appropriate postharvest fruit handling including cold storage [3]. Storage at low temperatures is a common practice in developed stone fruit producing countries since it effectively slows fruit ripening and retards the growth of the pathogens. However, although in general fungal decay is not an important problem during the cold storage period, it may become significant during subsequent fruit handling and marketing. Therefore, specific postharvest antifungal treatments are usually needed to prevent economic losses, especially on stone fruits for distant markets [5]. Postharvest treatments in the packinghouse with synthetic chemical fungicides are recommended when climatic conditions are favorable to the disease and field control practices are insufficient. The approved active ingredients (a.i.) depend on the producing country, with fludioxonil and fenhexamid being the compounds most commonly used [6]. In Spain, fludioxonil (Scholar 230 SC, 23% a.i., Syngenta, Madrid, Spain) is fully registered since 2016 for postharvest use on stone fruits after several special emergency authorizations by the Spanish Ministry of Agriculture starting in 2012. Moreover, pyrimethanil in a fumigant presentation (Deccopyr Pot, 30% a.i., Decco Iberica Post-Cosecha SAU, Paterna, Valencia, Spain) was registered in 2017 for postharvest gaseous treatment of stone fruits. Nevertheless, the use of conventional chemical fungicides raises important concerns about human health risks and environmental problems associated with chemical residues, as well as the proliferation of resistant biotypes of fungal pathogens that makes fungicides ineffective [7,8]. Therefore, there is an increasing interest to find and develop new treatments to replace postharvest conventional fungicides.

Different alternative treatments to synthetic fungicides to control postharvest diseases of stone fruit have been studied for many years, and the few of them that have been commercially implemented recently were mainly to control BR [3]. Alternative methods can be physical (mainly heat treatments), biological (use of antagonistic microorganisms as biocontrol agents), or low-toxicity chemicals [9]. Low-toxicity chemicals are natural or synthetic compounds with low toxicity to humans and wildlife. Among them, the use of food additives or generally recognized as safe (GRAS) substances is a relatively new trend for controlling plant pathogens as an alternative to synthetic fungicides. Many organic and inorganic salts are classified as food additives (E-number) by the European Food Safety Authority (EFSA) or as GRAS substances by the United States Food and Drug Administration (USFDA) [8]. These salts are extensively used as food additives (e.g., taste and pH regulation) on many agrifood applications, and some of them have antimicrobial activity [10]. Furthermore, they are inexpensive, easy to apply, and they exhibit a wide range of antimicrobial activity [11]. Due to these advantages, in recent years, the potential of GRAS salts such as some benzoates, carbonates, parabens, and sorbates, among others, to control several postharvest diseases has been tested on a variety of fresh fruits, either as aqueous solutions or as ingredients of edible coatings [12,13,14,15,16]. 

Some inorganic or organic salts were reported as partially effective in controlling postharvest diseases of peaches, nectarines, and plums when applied as aqueous solutions after harvest. In a large study conducted in California, more than 20 GRAS substances at concentrations between 20 and 400 mM were evaluated in in vivo primary screenings to control diseases caused by seven of the most important postharvest pathogens of stone fruits, including *M. fructicola*, *R. stolonifer*, *G. candidum*, *B. cinerea*, *P. expansum*, *Alternaria alternata* (Fr.: Fr.) Keissler, and *Mucor piriformis* E. Fischer [17]. Overall, the most effective but non-phytotoxic treatments were potassium sorbate (PS), sodium benzoate (SB), sodium sorbate (SS), sodium carbonate (SC), and potassium carbonate (PC). The salts PS and SB were also applied as 60 s dips in aqueous solutions at ambient temperature and significantly reduced BR on fruit artificially inoculated with *M. fructicola* [17]. Similarly, in a work conducted in Italy, PS-dips reduced significantly the incidence of BR caused by *M. laxa* on peaches and nectarines [18]. Other salts, such as sodium bicarbonate (SBC) and potassium bicarbonate (PBC), also showed potential to control postharvest decay of peaches, nectarines, and sweet cherries [19,20]. 

In view of the need for alternatives to chemical fungicides for postharvest disease control, interest has arisen to evaluate the performance of other food additives and GRAS salts for this purpose. Recent literature suggests that sulfur-containing salts, such as metabisulfites and aluminum-containing sulfates, are effective salts to control some important postharvest diseases of horticultural produce. Although sulfites (SO_3_^2−^), bisulfites (HSO_3_^−^), and metabisulfites (S_2_O_5_^2−^) have been sometimes linked to adverse allergies [21,22], the compounds in the sulfites group (E-numbers E 220-228) are currently approved as food additives for different uses on whole fresh fruits and vegetables [23]. Likewise, nowadays, the aluminum sulfates (E-numbers E 520-523) are considered of minimal human health concern in the regulated uses, after re-evaluation by the EFSA in 2018 [24]. Regarding agricultural uses, the control of gray mold caused by *B. cinerea* by postharvest sulfur dioxide (SO_2_) fumigations or in-package sodium metabisulfite pads is widely extended in the table grape industry [25,26]. These technologies have also been tested on figs [27] and blueberries [28].

Sulfur-containing salts have been tested both in vitro and in vivo against several pathogens of commercial importance on crops such as the potato, carrot, and citrus fruits [29,30,31,32,33,34,35], but, to our knowledge, these salts have not been evaluated for the control of major postharvest diseases of stone fruit. Therefore, the objectives of this study were to (i) evaluate the effect of various sulfur-containing salts, at different concentrations, on the in vitro mycelial growth of three major stone fruit postharvest pathogens (*M. fructicola*, *R. stolonifer*, and *G. candidum*) and (ii) determine the ability of the most promising salts and concentrations to control BR, SR, and RR on artificially inoculated nectarines. In vivo primary screenings were first performed, and, afterwards, selected compounds and doses were evaluated in small-scale dip treatments. 

## 2. Materials and Methods

### 2.1. Food Additives

Sodium metabisulfite (SMBS), potassium metabisulfite (PMBS), aluminum sulfate (AlS), and aluminum potassium sulfate (AlPS) were the sulfur-containing salts evaluated. They are classified as food additives within the European Union, with the respective E-numbers E-223, E-224, E-520, and E-522. Likewise, they are classified as GRAS compounds by the USFDA, with the respective subparts in the CFR Title 21: 182.3637, 182.3766, 182.1125, and 182.1129. Their most important physicochemical properties have been reported in Martínez-Blay et al. [32]. The food additives SMBS and AlS were purchased from Honeywell Research Chemicals Fluka (Seelze, Germany) and PMBS and AlPS from Acros Organics BVBA (Geel, Belgium). 

### 2.2. Fungal Pathogens

The autochthonous strains *M. fructicola* MeCV-2, *G. candidum* FH-ALP-1, and *R. stolonifer* FH-CV-1 were obtained from decayed stone fruits collected in the Valencia area (Spain), identified, and cultured at the IVIA-CTP. For the experiments, 7–14-day-old fungal cultures grown on potato dextrose agar (PDA, Scharlab S.L., Barcelona, Catalonia, Spain) Petri dishes at 25 °C were used. For in vitro studies, mycelial plugs from these cultures were extracted with a sterilized cork borer (5 mm in diameter). For in vivo trials, high density-conidial suspensions were prepared in Tween 80 in sterile water (0.05%, *w*/*v*; Panreac-Química S.A., Barcelona, Spain). After passing through two layers of cheesecloth, the density of the suspension was measured with a hemocytometer and diluted with sterile water to obtain an inoculum density of 1 × 10^3^ spores/mL for *M. fructicola* and *R. stolonifer* and 1 × 10^6^ spores/mL for *G. candidum*.

### 2.3. In Vitro Antifungal Activity

In vitro mycelial growth of *M. fructicola*, *G. candidum*, and *R. stolonifer* was evaluated on 90-mm plastic PDA Petri dishes. The medium was amended (at 40–45 °C) with sterile aqueous solutions of the respective GRAS salt to achieve final concentrations in the medium of 10, 20, 30, 50, and 100 mM. Afterwards, a 5 mm diameter plug of the corresponding fungal culture was inoculated in the center of each plate and incubated for up to 10 days at 25 °C in the dark in a growth cabinet. PDA plates not amended with any salt were used as controls. Five replicates (PDA dishes) were used for each pathogen, salt, and concentration. Radial mycelial growth on each plate was calculated as the mean of two perpendicular fungal colony diameters. Measurements were performed after 3, 5, 7, and 10 days for *M. fructicola* and *G. candidum*, and after 1, 2, and 3 days of incubation in the case of *R. stolonifer*. Results after 10 days for *M. fructicola* and *G. candidum* and after 3 days for *R. stolonifer* are presented as the percentage of mycelial growth inhibition with respect to control plates, calculated as described in Martínez-Blay et al. [32]. 

### 2.4. In Vivo Curative Activity

#### 2.4.1. Fruit

The experiments were conducted with nectarines (*Prunus persica* (L) Batsch var. *nucipersica* (Suckow) Schneid) cvs. ‘Luciflora’ (white flesh) and ‘Ambra’ (yellow flesh) obtained from orchards in Carlet (Valencia, Spain) and Turís (Valencia, Spain), respectively. Fruit not subjected to commercial postharvest treatments were purchased from local agricultural cooperatives in these locations and transported to the IVIA-CTP, where they were used the same day or stored until use (for up to 5 days) at 1 °C and 90% relative humidity (RH). Cold-stored fruit were removed from the cold room and held at room temperature for 18–24 h before the experiment. Prior to each experiment, fruit were selected, randomized, superficially washed, and disinfected by dipping them for 4 min in a diluted bleach solution (0.05% sodium hypochlorite), rinsed abundantly with tap water, and allowed to air-dry.

#### 2.4.2. Fungal Inoculation 

Selected and disinfected nectarines were wounded once in the equator using a stainless-steel rod with a probe tip 1 mm wide and 2 mm in length. Then, the wound was immediately inoculated, using a micropipette, with 20 µL of the spore suspension of the target pathogen prepared as described above. Fruit inoculated with *M. fructicola* or *G. candidum* were kept in a storage room at 20 °C for 24 h, until treatment. Due to the aggressiveness and rapid growth of *R. stolonifer*, fruit inoculated with this pathogen were treated when the inoculum drop was dried, about 2–3 h after inoculation. In all cases, the in vivo treatments consisted of, as described below, an assessment of curative activity, since the treatments were applied to fruit already infected by each fungus.

#### 2.4.3. In Vivo Primary Screenings 

The salts SMBS, PMBS, AlS, and AlPS were tested at several concentrations in simple and fast screening trials to control BR caused by *M. fructicola*, SR caused by *G. candidum*, and RR caused by *R. stolonifer* on ‘Luciflora’ nectarines previously inoculated with the pathogens. A 200-mM sterile mother solution of SMBS, PMBS, AlS, and AlPS was prepared, and sterile solutions at concentrations of 1, 5, 10, 50, and 100 mM SMBS, PMBS, AlS, and AlPS were prepared by diluting them with sterile water. On nectarines inoculated with *M. fructicola* or *G. candidum*, 30 µL of SMBS, PMBS, AlS, or AlPS aqueous solution were placed, with a micropipette, in the same inoculated peel wound about 24 h after the pathogen inoculation. Specifically, the four salts were tested at 1, 5, 10, 50, and 100 mM against BR and at 10, 50, and 100 mM against SR. On fruit inoculated with *R. stolonifer*, the same procedure was used, but the treatments were applied about 2–3 h after the inoculation of the pathogen. In all cases, control fruit were treated with 30 µL with sterile distilled water. A total of 4 replicates of 5 nectarines each were used for each combination of food additive, salt concentration, and pathogen. For *M. fructicola* and *G. candidum*, treated fruit were placed on plastic cavity sockets on cardboard trays and incubated at 20 °C and 90% RH for up to 8 days. In the case of *R. stolonifer*, treated fruit were placed on individual empty Petri dishes within closed plastic containers with wet paper towels on the bottom and small holes to allow aeration, and incubated at 20 °C and 90% RH for up to 4 days. 

#### 2.4.4. Dip Treatment Conditions

The most promising treatments selected from the in vivo primary screenings were tested in small-scale dip trials with ‘Ambra’ nectarines to evaluate the potential of dip applications as commercial treatments for decay control in stone fruit packinghouses. Fungal wound inoculations with *M. fructicola* (24 h before treatment) and *R. stolonifer* (2–3 h before treatment), both at a concentration of 10^3^ spores/mL, were performed as described above. Food additive treatments assayed to control BR were 1 min dips in aqueous solutions of SMBS, PMBS, AlS, or AlPS, all at 10 mM and ambient temperature (20 °C). Selected food additive treatments assayed to control RR were 1 min dips at 20 °C in aqueous solutions of AlS or AlPS, both at 50 mM. All dips were performed in stainless steel buckets containing 10 L of the corresponding salt solution. Fruit previously inoculated were completely immersed in the treatment solution for 1 min, and control fruit were dipped in water. Four replicates of 10 fruit each (40 fruit) per treatment were carried out. Fruit were arranged in plastic cavity sockets on cardboard trays (for BR experiments) or in closed plastic containers with humid paper towels (for RR experiments), and incubated at 20 °C for up to 8 days. Decay was assessed after 4, 6, and 8 days of incubation in the case of BR and after 3, 4, 5, and 6 days of incubation in the case of RR. These dip trials were conducted twice. 

#### 2.4.5. Disease and Phytotoxicity Assessments

After each incubation period, disease incidence (percentage of infected wounds), pathogen sporulation (percentage of fruit with visible spores), and disease severity were determined. For BR and SR, disease severity was measured as the lesion diameter, in mm. Due to the aggressiveness and rapid growth of *R. stolonifer*, a Rhizopus rot severity index (RRSI), based on an objective quantitative scale, was established in which each fruit was assigned into one of the following five categories: score = 0, no visible disease symptoms; score = 1, decay lesion < 25% of fruit surface; score = 2, 25% fruit surface < decay lesion < 50%; score = 3, 50% fruit surface < decay lesion < 75%; score = 4, decay lesion > 75% fruit surface. The RRSI only referred to the size of the lesions, not to the presence of external mycelium or spores.

In preliminary screenings, potential damage caused by the application of the treatment droplet on the peel tissue surrounding each wound was visually assessed at the end of the incubation period. In dip treatments, potential fruit peel phytotoxicity caused by the salts were also visually assessed following the scale: 1 = none, 2 = slight (lesions < 25% of the peel surface), 3 = moderate (25–50% of the peel), and 4 = severe (>50% of the peel).

### 2.5. Statistical Analysis

Data were analyzed by analysis of variance (ANOVA) with the software Statgraphics Centurion XVII (StatPoint Technologies Inc., Warrenton, VA, USA). Disease incidence and pathogen sporulation were calculated as percentages. These values were arcsine-transformed to improve the homogeneity of variances. In some cases, results are presented as percentages of reduction with respect to control fruit. Statistical significance was judged at the 95% level of confidence (*p* = 0.05). On repeated experiments, means from the different tests are presented since the factor ‘trial’ was not significant. Fisher’s Protected Least Significant Difference (LSD) was used as a means separation test. Shown values are non-transformed means. 

## 3. Results

### 3.1. Inhibition of In Vitro Mycelial Growth

Sulfur-containing salts were very effective, even at low concentrations, in inhibiting the mycelial growth on PDA plates of the three tested target pathogens (Table 1). After 10 days of incubation at 25 °C, all salts and concentrations completely inhibited the growth of *M. fructicola* and *G. candidum*, with the exception of AlPS at 10 and 20 mM, which inhibited these fungi by more than 65%. Similarly, after 3 days of incubation at 25 °C, *R. stolonifer* was completely inhibited by most salts and concentrations. Inhibition was only lower than 100% with AlS at 10 mM and AlPS at 10 and 20 mM.

### 3.2. In Vivo Curative Activity

#### 3.2.1. Primary Screenings

**Figure 1:**
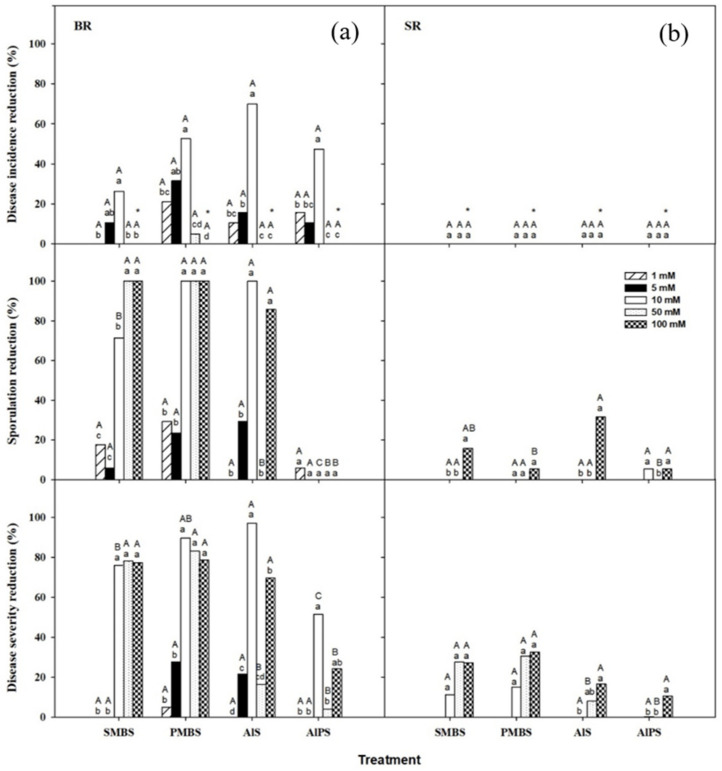
Curative activity of sodium metabisulfite (SMBS), potassium metabisulfite (PMBS), aluminum sulfate (AlS), and aluminum potassium sulfate (AlPS) at different concentrations against (**a**) brown rot (BR) and (**b**) sour rot (SR) in in vivo primary screenings with ‘Luciflora’ nectarines artificially inoculated with *Monilinia fructicola* and *Geotrichum candidum*, respectively, treated 24 h later, and incubated for 8 days at 20 °C. Five salt concentrations (1, 5, 10, 50, and 100 mM) were evaluated against BR, while three (10, 50, and 100 mM) were evaluated against SR. Disease reductions were determined with respect to control fruit treated with water, in which incidence, sporulation, and severity were respectively 95%, 85%, and 63.6 mm for BR and 100%, 95%, and 40.5 mm for SR. For each disease and dependent variable, columns with different capital letters indicate significant differences among salts for each concentration, and columns with different lowercase letters indicate significant differences among concentrations for each salt, according to Fisher’s protected LSD test (*p* < 0.05) applied after an ANOVA. Incidence and sporulation values were arcsine-transformed. Non-transformed means are shown. * indicates treatments that caused visible peel phytotoxicity.

The influence of the concentration of SMBS, PMBS, AlS, and AlPS on the development of BR, SR, and RR was evaluated in in vivo primary screenings. Tested concentrations were 1, 5, 10, 50, and 100 mM for BR and 10, 50, and 100 mM for SR and RR. The treatments AlS, AlPS, PMBS, and SMBS at 10 mM were the most effective ones, reducing the incidence of BR by 70, 48, 53, and 32%, respectively, in comparison to water-treated control fruit (Figure 1a). These treatments also considerably reduced BR severity on artificially inoculated nectarines, and severity reductions of 90 and 97% were achieved with 10 mM PMBS and AlS, respectively. Moreover, PMBS and AlS at 10 mM completely inhibited the sporulation of *M. fructicola* (Figure 1a).

However, none of the salts reduced SR incidence; only the highest concentrations of SMBS and PMBS were able to reduce its severity, and then by only 25–30% (Figure 1b). AlS at 100 mM was the only treatment that reduced the sporulation of *G. candidum* by more than 30% (Figure 1b). Therefore, further in vivo research with these salts for SR control was not considered. Conversely, the four salts at their lowest most effective concentration (10 mM) were selected to be evaluated as dip treatments for potential commercial BR control. 

**Figure 2:**
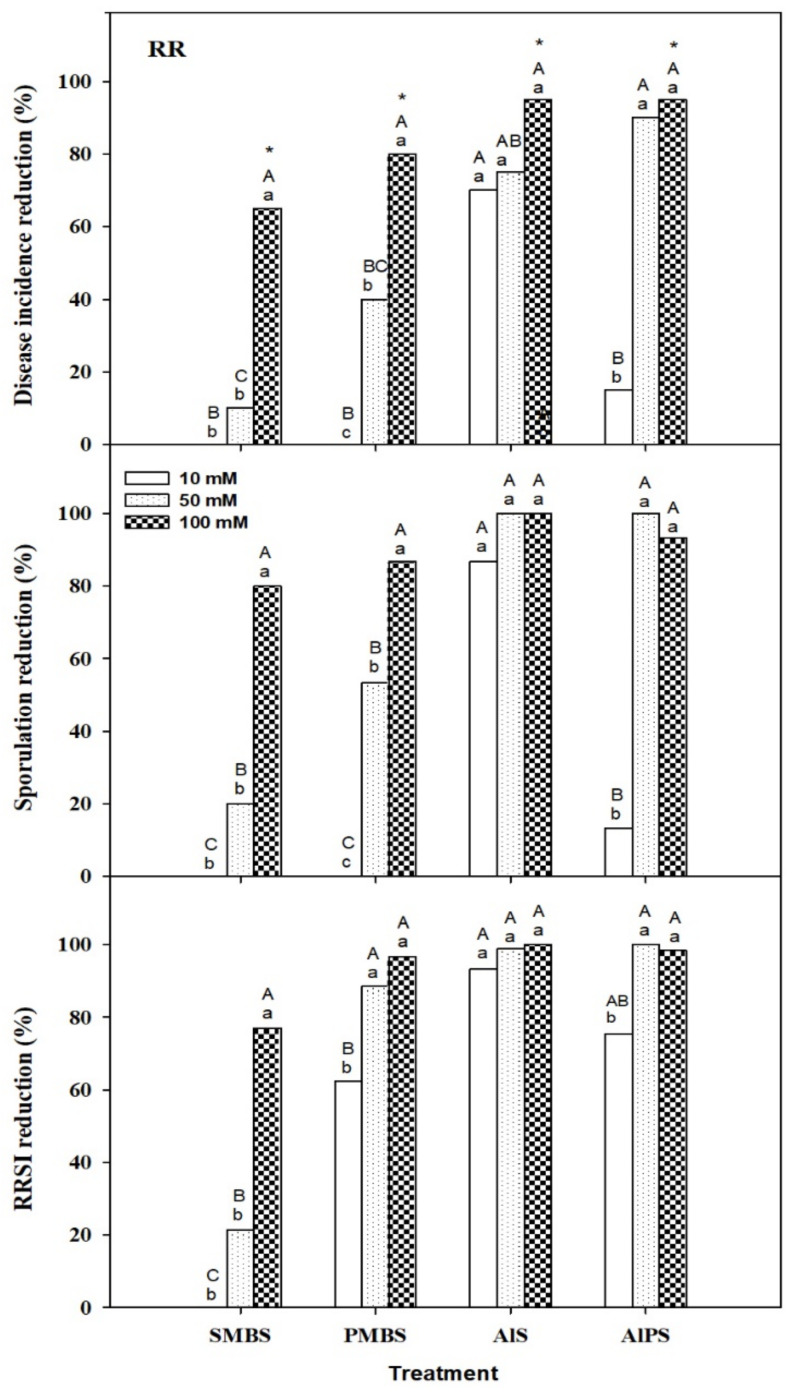
Curative activity of sodium metabisulfite (SMBS), potassium metabisulfite (PMBS), aluminum sulfate (AlS), and aluminum potassium sulfate (AlPS) at different concentrations (10, 50, and 100 mM) against Rhizopus rot (RR) in in vivo primary screenings with ‘Luciflora’ nectarines artificially inoculated with *Rhizopus stolonifer*, treated 2–3 h later, and incubated for 4 days at 20 °C. Reductions in disease incidence, pathogen sporulation, and Rhizopus rot severity index (RRSI, score 0–4) were determined with respect to control fruit treated with water, in which incidence, sporulation, and RRSI were respectively 100%, 100%, and 3.1. For each dependent variable, columns with different capital letters indicate significant differences among salts for each concentration and different lowercase letters indicate significant differences among concentrations for each salt (Fisher’s protected LSD test, *p* < 0.05, after an ANOVA). Incidence and sporulation were arcsine-transformed. Non-transformed means are shown. * indicates treatments that caused visible peel phytotoxicity.

Among all the salts and concentrations tested to control RR, AlS and AlPS, both at 50 or 100 mM, were the most effective after 4 days of incubation at 20 °C, with reductions in incidence, sporulation, and RRSI of 90–100% (Figure 2). SMBS and PMBS at 100 mM also reduced satisfactorily disease development, with incidence reductions of 65–80%, although this control ability was significantly lower than that of AlS and AlPS, even at lower concentrations. PMBS and especially SMBS at 10 or 50 mM lack curative activity against RR (Figure 2). Therefore, AlS and AlPS at their lowest most effective concentration of 50 mM were selected as treatments to be evaluated in dip treatments for RR control.

The application of the four salts at 100 mM caused apparent phytotoxicity, with darkening and sinking on the fruit peel at the inoculation point (Figure 1). Nevertheless, no damage was caused by the application of the droplet of each chemical solution on the peel tissue surrounding the wound at the concentrations of 1, 5, 10, and 50 mM. 

#### 3.2.2. Dip Treatments 

**Figure 3:**
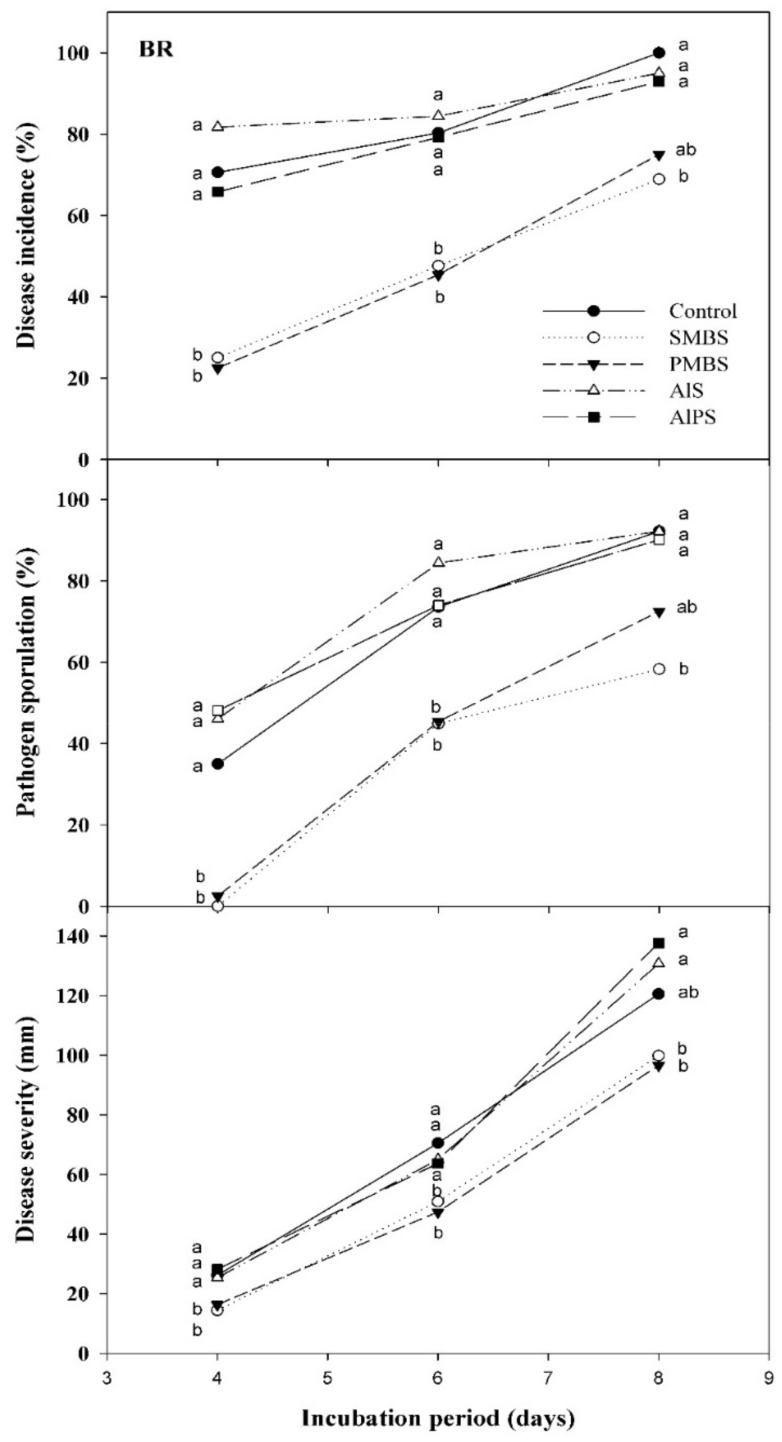
Curative activity of 1 min dips at 20 °C in water (control) or aqueous solutions of sodium metabisulfite (SMBS), potassium metabisulfite (PMBS), aluminum sulfate (AlS), and aluminum potassium sulfate (AlPS) at 10 mM to control brown rot (BR) on ‘Ambra’ nectarines artificially inoculated with *Monilinia fructicola*, dipped 24 h later, and incubated for up to 8 days at 20 °C. Disease incidence and severity and pathogen sporulation are presented. For each dependent variable and evaluation date, means with different letters are significantly different according to Fisher’s protected LSD test (*p* < 0.05) applied after an ANOVA. Represented values are means from two repeated experiments. Incidence and sporulation values were arcsine-transformed. Non-transformed means are shown.

After incubation at 20 °C for up to 8 days, SMBS and PMBS, at 10 mM, were clearly superior to AlS and AlPS to reduce BR on ‘Ambra’ nectarines dipped for 1 min in aqueous solutions of the salts (Figure 3). 

After 4 days of incubation, disease incidences in fruit immersed in 10 mM SMBS or PMBS were about 20–25%, while it was about 70% on control fruit (about 70% BR incidence reduction). However, the control ability decreased with incubation time, and after 8 days, disease incidence reductions with respect to the control on fruit immersed in these salt solutions were 25–30%. Disease reductions on nectarines dipped in AlS or AlPS solutions were not significant during the entire incubation period. Likewise, a similar pattern was observed for pathogen sporulation and BR severity, which were significantly reduced by SMBS and PMBS dips, but not by AlS or AlPS (Figure 3). 

**Figure 4:**
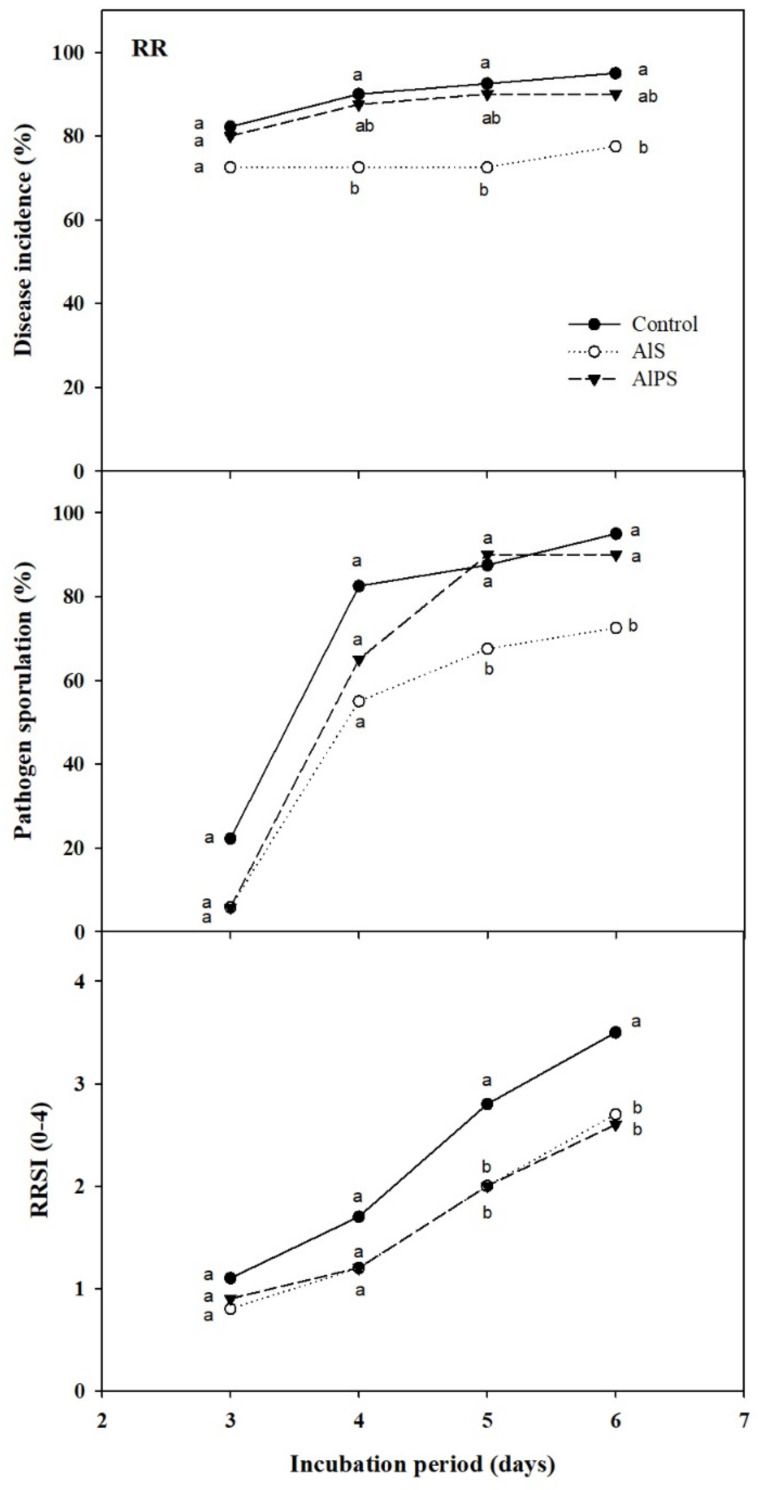
Curative activity of 1 min dips at 20 °C in water (control) or aqueous solutions of aluminum sulfate (AlS) and aluminum potassium sulfate (AlPS) at 50 mM against Rhizopus rot (RR) on ‘Ambra’ nectarines artificially inoculated with *Rhizopus stolonifer*, dipped 2–3 h later, and incubated for up to 6 days at 20 °C. Disease incidence, Rhizopus rot severity index (RRSI, score 0–4), and pathogen sporulation are presented. For each dependent variable and evaluation date, means with different letters are significantly different according to Fisher’s protected LSD test (*p* < 0.05) applied after an ANOVA. Represented values are means from two repeated experiments. Incidence and sporulation values were arcsine-transformed. Non-transformed means are shown.

On dip trials to control RR, AlS at 50 mM was significantly superior to AlPS at the same concentration to reduce disease incidence and pathogen sporulation on ‘Ambra’ nectarines wound-inoculated with *R. stolonifer* and dipped for 1 min in aqueous solutions of the salts (Figure 4). However, the effectiveness of this salt was low and RR incidence after 3 days of incubation at 20 °C was already around 70%. After 6 days, while disease incidence reached values of 75% on AlS-treated nectarines, it was around 95% on control and AlPS-treated fruit (reduction of 20% with respect to the control; Figure 4). In contrast, both salts equally reduced the RRSI under the experimental conditions, and, after 6 days, the average RRSI score was around 2.5 in comparison to values of 3.5 on water-treated fruit (Figure 4). 

After all dip treatments, no peel phytotoxicities were observed (injury scale value = 1).

## 4. Discussion

The activity of four sulfur-containing salts (SMBS, PMBS, AlS, and AlPS) in reducing the mycelial growth of *M. fructicola*, *G. candidum*, and *R. stolonifer* and, afterwards, their ability to control BR, SR, and RR of stone fruits were evaluated in the present study. In a first set of in vitro experiments, the mycelial growth of the three target pathogens on PDA plates was completely or largely inhibited by the four salts, indicating a considerable direct antifungal activity of these food additives. The important antifungal in vitro activity of sulfur-containing salts has been previously reported for different postharvest pathogens affecting important crops. For example, SMBS and PMBS, both at 10 mM, completely inhibited the mycelial growth of *Alternaria solani* Sorauer, *B. cinerea*, and *R. stolonifer* (pathogens causing decay of eggplant, pepper, and tomato, among other horticultural products), *Fusarium sambucinum* Fuckel (causing potato dry rot), and *Pythium sulcatum* R.G. Pratt & J.E. Mitch. (causing carrot cavity spot) [30]. Similarly, in another study by the same authors, the salt AlS at 1 mM inhibited the mycelial growth of *A. solani*, *B. cinerea*, *F. sambucinum*, and *R. stolonifer* and reduced above 60% the development of *P. sulcatum*, whereas AlPS reduced by more than 80% the mycelial growth of *B. cinerea* [31]. Other economically important potato pathogens, such as *A. alternata*, *Phytophthora infestans* (Mont.) de Bary, and *Fusarium solani* var. *coeruleum* (Sacc.) Booth were also inhibited by SMBS at the concentrations of 2, 20, and 2000 mM, whereas AlS reduced their growth by more than 70% [34]. Moreover, we observed in previous research that the four salts completely inhibited the development in PDA dishes of the important citrus pathogens *Penicillium digitatum* (Pers.:Fr.) Sacc, *Penicillium italicum* Wehmer, and *Geotrichum citri-aurantii* (Ferraris) Butler at concentrations of 30 mM or higher, and reduced it by more than 70% at lower doses [32]. To our knowledge, herein we first report the in vitro antifungal effect of SMBS, PMBS, AlS, and AlPS against *M. fructicola* and *G. candidum*.

In a second set of experiments, comprising in vivo primary screenings, the four salts, at various concentrations, were able to reduce BR significantly. Conversely, none of the salts and concentrations tested was able to control SR. In the case of RR, significant reductions in incidence and RRSI were achieved with AlS and AlPS, but not with SMBS and PMBS. In all screenings, only the highest salt concentration of 100 mM was phytotoxic and caused peel injury. According to these results, only effective and non-phytotoxic salts and concentrations were selected for efficacy evaluation in a third set of experiments consisting of dip treatments in small-scale trials. Results from these dip treatments showed that the food additives SMBS and PMBS were clearly superior to AlS and AlPS to control BR. On the other hand, AlS was significantly superior to AlPS to control RR, although the effectiveness of this salt was low. Overall, the effectiveness of the additives in in vivo primary screenings (drop of salt aqueous solution applied to the infected peel wound) was higher than that of dip treatments (immersion of infected fruit for 1 min in aqueous solutions of the salts), which perhaps can be explained by the shorter contact time of the infected fruit with the solution active ingredient.

Some inorganic or organic salts, such as benzoates, sorbates, and carbonates, have been previously reported as effective to some extent in controlling major postharvest diseases of stone fruit [17,19,20,36,37]. Moreover, sulfur-containing salts have been tested both in vitro and in vivo against several pathogens of commercial importance in crops such as the potato, carrot, and citrus [29,30,31,32,33,34,35], but, to our knowledge, this is the first work evaluating its use for the control of major postharvest diseases of stone fruits. Thus, the present paper is the first report on the potential curative activity of metabisulfite-containing salts, namely SMBS and PMBS, to control BR. Furthermore, the effectiveness of other sulfate-containing salts has also been reported. For example, AlS and AlPS were able to control postharvest diseases such as dry rot and cavity spots in potatoes and carrots, respectively [30,31]. However, in the present work, AlS and AlPS only provided a significant reduction in disease incidence and severity in the in vivo preliminary screenings against RR. Moreover, our negative results to control disease caused by *G. candidum* are in agreement with previous results obtained with another species of *Geotrichum*, namely *G. citri-aurantii* infecting citrus fruits [32,38].

The present results also show that the mode of action of dip treatments with SMBS, PMBS, AlS, and AlPS is rather fungistatic than fungicidal, since disease incidence and severity on treated nectarines increased with time during the incubation period, showing a low persistence of the salts. This was an expected result, as it has been generally reported for low toxicity compounds, including food additives and GRAS substances [10]. Besides, it is known that the ability of these salts to control postharvest diseases of fresh fruit cannot be predicted by their inhibitory potential in in vitro experiments. Typically, in vivo tests are a relevant procedure for selecting salts with high antifungal activity and potential for actual disease control. However, it is frequent to obtain inconsistent or inferior results when applied to infected fresh fruit in in vivo tests. This has been observed in the present work and is in agreement with previous research conducted with stone fruits [13,17,36]. Disease development is the result of a complex interaction among the host, the pathogen, and the environment. Interactions among the salt solution and fruit peel constituents may alter the environmental conditions in the wounds occupied by the fungus (e.g., pH, water activity), modifying the toxicity shown by the salt in a growing artificial medium and its effect on fungal growth and development [8,30,32]. Moreover, due to the nature of those interactions and the fungistatic nature of the activity of low-toxicity compounds such as GRAS salts (which lack or insufficient direct fungicidal action, in contrast with conventional synthetic fungicides), the level of disease reduction by this type of chemicals is strongly dependent not only on the target pathogen, but also on the fruit species, cultivar, and even fruit physical and physiological condition [17,31,33]. According to this, many factors can influence the mode of action of sulfur-containing fungistatic salts. For example, the pH of the solutions cannot explain by itself the differences in efficacy, as it has been the same for each solution tested, and different levels of control against the three postharvest diseases have been obtained. However, it has been reported that as a way to increase its pathogenicity, some fungal pathogens are able to alkalinize the medium in the peel infection wound, such as *Colletotrichum* spp. or *Alternaria* spp., whereas other species, such as *Penicillium* spp., acidify the ambient [39,40,41]. This mechanism promotes the production of polygalacturonases, hydrolytic enzymes that help to degrade the host cell walls, thus enhancing fungal colonization [42,43]. Moreover, sulfite salts in aqueous solutions are present as different compounds, existing a balance among them dependent on the pH [44]. For example, a percentage of sulfite salts is converted to sulfur dioxide when dissolved in water, and the accumulation of this substance in the cytoplasm has been cited to interfere with cellular components and processes, thus affecting pathogen development [45]. In fact, Avis et al. [46,47] found that the toxicity of SMBS against *F. sambucinum* and other fungi was due to lipid peroxidation of unsaturated fatty acids that caused disintegration of cell membranes. Besides, Smilanick et al. [48] observed that a narrow range of pH values greatly influenced the degree of toxicity of sulfite salts against *B. cinerea* and suggested that sulfur dioxide was responsible for the antimicrobial activity, increasing as the pH of the environment declined. 

Considering this information, the significant effect of pH on the action mode of sulfite and sulfate salts is clear [44,48]. Therefore, it is likely that the effectiveness of the salts SMBS, PMBS, AlS, and AlPS will increase if the pH is lower within the infected fruit peel wounds. Although the pH of the flesh of peaches and nectarines is acidic (generally in the range 3.5–5.0, depending on the type of flesh (yellow or white) and the particular cultivar), which favors the antifungal activity of these salts, the acidification or alkalinization effect derived from the fungal attack could increase or reduce this activity. To our knowledge, no information is available on the acidification capacity of *G.* *candidum*, *M. fructicola*, and *R. stolonifer* in stone fruits. As a conjecture to explain the present results, it can be proposed that significant differences in the acidification degree of the medium may enhance the effectiveness of SMBS and PMBS to control BR in comparison with SR and RR, as well as contribute to the different effectiveness of AlS and AlPS to control RR in comparison with BR and SR. In any case, for now, the action mechanism of these four salts against major stone fruit postharvest diseases is not completely understood, and future studies are needed to elucidate why these salts better controlled disease caused by *M. fructicola* than disease caused by *G. candidum* or *R. stolonifer*.

Besides the limited direct fungicidal activity against the tested pathogens, another factor possibly explaining the general lack of disease control obtained with AlS and AlPS could be the lack of induction of biochemical defenses by the treated fruit host, which has been previously reported as one of the main mechanisms of action of aluminum containing salts [31,33,49,50]. Other important factors that may have affected disease control under our experimental conditions are likely the increased contact time of the salt solution drop with the peel wound in the in vivo primary screenings in comparison to the shorter contact time in small-scale dip treatments, the penetration capability of the active ingredient into peel wounds, and the different nectarine cultivars used in the assays. 

Overall, this study has revealed that SMBS, PMBS, AlS, and AlPS have potent in vitro antifungal activity at relatively low concentrations (10 mM) against major stone fruit postharvest pathogens. However, this does not correlate with disease control ability in vivo, and the salts did not satisfactorily reduce SR and RR, and only treatments with SMBS and PMBS showed some potential for BR control, since they significantly reduced BR incidence and severity and sporulation of *M. fructicola* on infected nectarines. Although dip treatments were rather fungistatic, and their effectiveness and persistence were not comparable to those provided by postharvest treatments with synthetic fungicides, we believe it is important in the current context to identify potential non-polluting tools to be used in commercial integrated disease management programs. Further research can be focused on the improvement in the efficacy by means of a combination of the most effective sulfur-containing food additives, such as SMBS and PMBS at different concentrations, or an integration of these salt treatments with other alternative control methods.

Sulfur salts have a wide range of agricultural and environmental uses, as they are currently considered safe for human ingestion when applied at suitable concentrations [23,51,52]. These food additives are used both in the food processing industry (e.g., wines and some liquors) and the fresh fruit industry, specifically as postharvest treatments for grapes (sulfur dioxide fumigations or in-package sodium metabisulfite pads) for the control of gray mold caused by *B. cinerea* [51,53]. Novel potential uses of sulfur salts as non-polluting alternatives to synthetic fungicides were evaluated in this research work. Results showed that treatments with SMBS and/or PMBS aqueous solutions might be included in integrated management programs for the control of BR, although not for the control of SR and RR on nectarines or peaches.

## 5. Conclusions

This research represents a first step into the uses of metabisulfite salts to control major stone fruit postharvest fungal diseases. However, further studies are needed to improve the suitability of these treatments, to evaluate their level of disease control on other commercially important peach and nectarine cultivars and more stone fruit species such as plums and cherries, and to assess their effect on the physicochemical and sensorial attributes of long-term cold-stored fruit.

## Figures and Tables

**Table 1:** Inhibition of mycelial growth (%) of stone fruit postharvest pathogens on PDA Petri dishes amended with sulfur-containing food additives at several concentrations and incubated at 25 °C.

Food Additive ^b^	Concentration (mM)	Inhibition of Mycelial Growth ^a^ (%)		
		*Monilinia fructicola* ^c^	*Geotrichum candidum* ^c^	*Rhizopus stolonifer* ^c^
SMBS	10	100 a	100 a	100 a
	20	100 a	100 a	100 a
	30	100 a	100 a	100 a
	50	100 a	100 a	100 a
	100	100 a	100 a	100 a
PMBS	10	100 a	100 a	100 a
	20	100 a	100 a	100 a
	30	100 a	100 a	100 a
	50	100 a	100 a	100 a
	100	100 a	100 a	100 a
AlS	10	100 a	100 a	78.95 d
	20	100 a	100 a	100 a
	30	100 a	100 a	100 a
	50	100 a	100 a	100 a
	100	100 a	100 a	100 a
AlPS	10	80.47 c	65.47 b	82.56 c
	20	86.86 b	100 a	90.47 b
	30	100 a	100 a	100 a
	50	100 a	100 a	100 a
	100	100 a	100 a	100 a

^a^ Mean of two perpendicular diameters of the fungal colony on 5 replicate dishes per fungus, salt, and concentration. For *M. fructicola* and *G. candidum*, mycelial growth was determined after 10 days and for *R. stolonifer* after 3 days of incubation at 25 °C. After this period, colony diameter in control plates was the maximum (86 mm) for all three pathogens. ^b^ SMBS: sodium metabisulfite, PMBS: potassium metabisulfite, AlS: Aluminum sulfate, AlPS: Aluminum potassium sulfate. ^c^ Within a column, values followed by the same letter are not significantly different according to Fisher‘s protected LSD test (*p* = 0.05).

## Data Availability

The data presented in this study belong to the IVIA and are available on request from the corresponding author.
